# Adaptive Parameter Modulation of Deep Brain Stimulation Based on Improved Supervisory Algorithm

**DOI:** 10.3389/fnins.2021.750806

**Published:** 2021-09-16

**Authors:** Yulin Zhu, Jiang Wang, Huiyan Li, Chen Liu, Warren M. Grill

**Affiliations:** ^1^Department of Biomedical Engineering, Duke University, Durham, NC, United States; ^2^School of Electrical and Information Engineering, Tianjin University, Tianjin, China; ^3^School of Automation and Electrical Engineering, Tianjin University of Technology and Education, Tianjin, China

**Keywords:** Parkinson’s disease, feedback signal, beta power, RBF neural network, supervisory control algorithm

## Abstract

Clinically deployed deep brain stimulation (DBS) for the treatment of Parkinson’s disease operates in an open loop with fixed stimulation parameters, and this may result in high energy consumption and suboptimal therapy. The objective of this manuscript is to establish, through simulation in a computational model, a closed-loop control system that can automatically adjust the stimulation parameters to recover normal activity in model neurons. Exaggerated beta band activity is recognized as a hallmark of Parkinson’s disease and beta band activity in model neurons of the globus pallidus internus (GPi) was used as the feedback signal to control DBS of the GPi. Traditional proportional controller and proportional-integral controller were not effective in eliminating the error between the target level of beta power and the beta power under Parkinsonian conditions. To overcome the difficulties in tuning the controller parameters and improve tracking performance in the case of changes in the plant, a supervisory control algorithm was implemented by introducing a Radial Basis Function (RBF) network to build the inverse model of the plant. Simulation results show the successful tracking of target beta power in the presence of changes in Parkinsonian state as well as during dynamic changes in the target level of beta power. Our computational study suggests the feasibility of the RBF network-driven supervisory control algorithm for real-time modulation of DBS parameters for the treatment of Parkinson’s disease.

## Introduction

Parkinson’s disease (PD) is a progressive neurodegenerative disorder resulting from death of dopaminergic neurons in the substantia nigra (Titcombe et al., 2001; Novikova et al., 2006; Bras et al., 2008; Jankovic, 2008; de Paor and Lowery, 2009). Deep brain stimulation (DBS), that delivers high-frequency electrical pulses via an implanted pulse generator to focal targets in the basal ganglia (BG) including the subthalamic nucleus (STN), the globus pallidus internus (GPi), or the ventrolateral thalamus (Vim), is a widely used therapy for treating PD when drug therapy such as the administration of levodopa no longer provides adequate control of symptoms (Haeri et al., 2005; Kiss et al., 2007; Vidailhet et al., 2007; Mehta and Sethi, 2009; Follett et al., 2010; Santaniello et al., 2011). Present open-loop DBS delivers invariant stimulation with parameters selected manually based solely on previous empirical evidence. Pre-programmed stimulation is applied regardless of changes in the patient’s clinical symptoms or underlying physiological activity, and open-loop DBS is limited in terms of efficacy, side effects and efficiency (Modolo et al., 2012; Popovych and Tass, 2012; Priori et al., 2012).

Optimization of stimulation parameters according to the individual and time-varying needs of patients is necessary to improve the treatment of PD (Androulidakis et al., 2008; Steiner et al., 2017). Several studies suggested that closed-loop DBS is an effective approach to improve therapeutic efficacy while limiting side effects and prolonging battery life (Doshi et al., 2003; Rosin et al., 2011; Little et al., 2013; Wu et al., 2015). Inspired by successful clinical use of closed-loop stimulation based on ECoG recordings in the treatment of epilepsy, this approach was initially adopted for DBS parameter modulation where the stimulation signal was switched on when beta oscillatory power exceeded a pre-set threshold in a primate model of PD (Little et al., 2013) and was subsequently extended to a dual threshold algorithm (Velisar et al., 2019). The design of closed-loop DBS, which uses a feedback signal and real-time adjustment of stimulation parameters, is considered the next frontier in the field of neuromodulation (Pizzolato and Mandat, 2012; Broccard et al., 2014; Hebb et al., 2014; Arlotti et al., 2016a; Swann et al., 2018).

A range of challenges are associated with closed-loop DBS including detectable control signals that are stable and robust in the long term (Little and Brown, 2012; Hoang et al., 2017; Steiner et al., 2017), understanding the relationship between patient states and brain control signals (Buzsáki et al., 2012), closed-loop control algorithms for automatic adjustment of stimulation parameters (Pirini et al., 2009; Guo and Rubin, 2011; Gorzelic et al., 2013), and comparisons of open-loop versus closed-loop DBS and clarification of their underlying mechanisms (Parastarfeizabadi and Kouzani, 2017). Therefore, the objective of this manuscript is to develop a computational model-based closed-loop scheme to adjust automatically the stimulation parameters for suppressing abnormal oscillatory activity in the BG. Local field potential (LFP) signals directly recorded from the DBS electrode appear to be a promising source of feedback signals (Mazzoni et al., 2015), and beta-band oscillations in the LFP are related to bradykinesia and rigidity in persons with PD (Beudel et al., 2017; Rosa et al., 2017; Deffains and Bergman, 2019; Lofredi et al., 2019; Montgomery, 2020). Here, a biophysically-based computational network model serves as the plant for the design of closed-loop control systems, from which LFP signals are obtained to simulate clinically detectable and recordable signals.

The highly nonlinear dynamics of the cortex-basal ganglia-thalamus network make the selection of controller parameters a substantial challenge (Kumaravelu et al., 2016). For traditional proportional-integral-derivative (PID) control, it is difficult to select appropriate controller gains, and the dependence on the precise mathematical model of the plant means that control accuracy cannot be guaranteed (Su et al., 2019). Neural network control has several potential advantages in this application. First, the capacity of neural network controllers to represent arbitrary functions avoids the complex mathematical analysis required for traditional adaptive control theory. In addition to modeling the complex and non-linear plant, neural networks can also act as the controller and continuously adjust the internal connection weights according to learning rules to minimize a given performance index. Thus, a supervisory control method based on radial basis function (RBF) neural networks was developed in this manuscript. In section “Materials and Methods,” we introduce the feedback signal selected for closing the loop of DBS and detail the design of the closed-loop control system. The control effects of a traditional controller and the intelligent supervisory controller are analyzed and compared in section “Results,” and the results are discussed in section “Discussion.” The proposed algorithm adaptively produced effective stimulation signals in response to changes in the state (plant) and the reference (target) signal.

## Materials and Methods

### Cortical-Basal Ganglia-Thalamus Network Model

**FIGURE 1:**
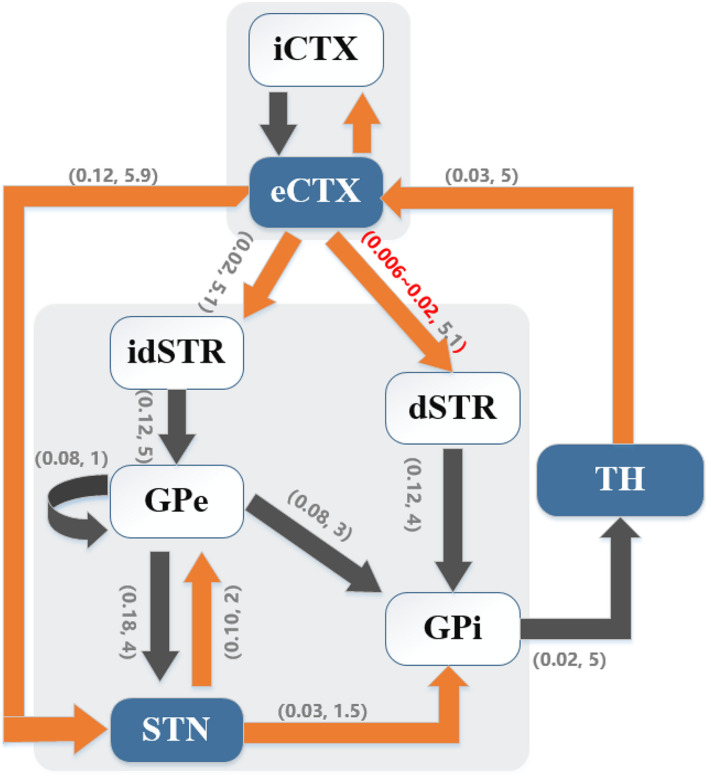
Cortical-basal ganglia-thalamus network model. Gray blocks represent the collections of cortical (CTX) neurons and basal ganglia (BG) neurons, respectively. Model schematic shows connections within the network, where black lines denote inhibitory connections and orange lines denote excitatory connections. Here, the direct pathway (eCTX → dSTR → GPi → TH → eCTX), the indirect pathway (eCTX → idSTR → GPe → GPi → TH → eCTX) and the hyper-direct pathway (eCTX → STN → GPi → TH → eCTX) are depicted. As well, excitatory-inhibitory coupling exists between STN and GPe. Excitatory eCTX and inhibitory iCTX neurons also receive synaptic connections from each other. The numbers in parentheses on the arrows indicate the synaptic conductance (*mS*/*cm*^2^) and transmission delay (ms), respectively.

A biophysically-based model of the cortex-basal ganglia-thalamus network (Kumaravelu et al., 2016), modified from the original Rubin-Terman model (Rubin and Terman, 2004), was adopted as a platform to develop and evaluate the controllers. The model included representations of neurons in cortex (CTX), striatum (STR) [sum of direct striatum (dSTR) and indirect striatum (idSTR)], STN, globus pallidus (GP) (sum of externa part GPe and interna part GPi) and thalamus (TH), and all the nuclei were interconnected through either excitatory or inhibitory synaptic connections to form a network. Each nucleus contained 10 single-compartment model neurons (Figure 1).

The neurons of the STN, GP, TH, and STR were modeled using Hodgkin-Huxley (HH) type equations:


(1) $$\begin{array}{c} C\,\frac{\text{d}\,v_{\text{STN}}}{\text{d}\,t}=-I_{\text{Na}}-I_{\text{K}}-I_{\text{l}}-I_{\text{T}}-I_{\text{CaK}}-I_{\text{a}}-I_{\text{L}}-I_{\text{syn}}\\ C\,\frac{\text{d}\,v_{\text{GP}}}{\text{d}\,t}=-I_{\text{Na}}-I_{\text{K}}-I_{\text{l}}-I_{\text{T}}-I_{\text{Ca}}-I_{\text{ahp}}-I_{\text{syn}}+I_{\mathrm{app}\,\_\,\mathrm{GP}}\\ C\,\frac{\text{d}\,v_{\text{TH}}}{\text{d}\,t}=-I_{\text{Na}}-I_{\text{K}}-I_{\text{l}}-I_{\text{T}}-I_{\text{syn}}+I_{\mathrm{app}\,\_\,\mathrm{TH}}\\ C\,\frac{\text{d}\,v_{\text{STR}}}{\text{d}\,t}=-I_{\text{l}}-I_{\text{K}}-I_{\text{Na}}-I_{\text{m}}-I_{\text{syn}} \end{array},$$

where *C* represented the membrane capacitance and was set to 1μ*F*/*cm*^2^ for all cell models, *v*_*i*_(*i* ∈ {*STN*,*GP*,*TH*,*STR*}) represented the transmembrane potential of the corresponding model neuron and was expressed in mV. *I*_Na_,*I*_K_,and*I*_l_ were the sodium current, potassium current and non-specific leak current, *I*_L_,*I*_T_,and*I*_Ca_ were, respectively, the L-type, T-type, and high-threshold calcium current, and *I*_a_,*I*_*m*_,*I*_Cak_,and*I*_AHP_ were, respectively, A-type, outward M-type potassium, calcium-dependent and after-threshold potassium current. Parameters and equations of the ionic currents are provided in Table 1. Therein, *a*, *b*, *c*, *d*1, *d*2, *h*, *m*, *n*, *p*, *q*, and *r* were activation or inactivation variables, and the gating kinetics took the form


(2) $$\frac{\text{d}\,X}{\text{d}\,t}=\frac{\lambda_{X}\,\left(X_{\infty }-X\right)}{\tau_{X}},$$

**TABLE 1:** Equations and parameters for subthalamic nucleus (STN), globus pallidus (GP), TH, and striatum (STR) model neurons.

STN	
Ionic currents	*I*_Na_ = 49*m*^3^*h*(*v*−60), *I*_*K*_ = 57*n*^4^(*v* + 90), *I*_*l*_ = 0.35(*v* + 60), *I*_*T*_ = 5*p*^2^*q*(*v*−165) *I*_CaK_ = *r*^2^(*v* + 90), *I*_*a*_ = 5*a*^2^*b*(*v* + 90), *I*_*L*_ = 15*c*^2^*d*_1_*d*_2_(*v*−165)
Gating kinetics	λ_*m*_ = 1,*w*_*m*_ = 40,σ_*m*_ = 8,τ_*m*_ = 0.2 + 3/(1 + *exp*⁡((*v* + 53)/0.7)) λ_*h*_ = 1,*w*_*h*_ = 45.5,σ_*h*_ = −6.4,τ_*h*_ = 24.5/(*exp*⁡((*v* + 50)/15) + *exp*⁡((*v* + 50)/16)) λ_*n*_ = 1,*w*_*n*_ = 41,σ_*n*_ = 14,τ_*n*_ = 11/(*exp*⁡(−(*v* + 40)/14) + *exp*⁡(−(*v* + 40)/50)) λ_*p*_ = 1,*w*_*p*_ = 56,σ_*p*_ = 6.7,τ_*p*_ = 5 + 0.33/(*exp*⁡((*v* + 27)/10) + *exp*⁡(−(*v* + 102)/15)) λ_*q*_ = 1,*w*_*q*_ = 85,σ_*q*_ = −5.8,τ_*q*_ = 400/(*exp*⁡((*v* + 50)/15) + *exp*⁡(−(*v* + 50)/16)) λ_*r*_ = 1,*w*_*r*_ = −0.17,σ_*r*_ = 0.08,τ_*r*_ = 2 λ_*a*_ = 1,*w*_*a*_ = 45,σ_*a*_ = 14.7,τ_*a*_ = 1 + 1/(1 + *exp*⁡((*v* + 40)/0.5)) λ_*b*_ = 1,*w*_*b*_ = 90,σ_*b*_ = −7.5,τ_*b*_ = 200/(*exp*⁡((*v* + 60)/30) + *exp*⁡(−(*v* + 40)/10)) λ_*c*_ = 1,*w*_*c*_ = 30.6,σ_*c*_ = 5,τ_*c*_ = 45 + 10/(*exp*⁡((*v* + 27)/20) + *exp*⁡(−(*v* + 50)/15)) λ_*d*1_ = 1,*wd*_1_ = 60,σ_*d*1_ = −7.5,τ_*d*_1__ = 400 + 500/(*exp*⁡((*v* + 40)/15) + *exp*⁡(−(*v* + 20)/20)) λ_*d*2_ = 1,*w*_*d*2_ = −0.1,σ_*d*2_ = −0.02,τ_*d*2_ = 130

**GP**	

Ionic currents	$I_{\text{Na}}=120\,m_{\infty }^{3}\,h\,\left(v-55\right)$, *I*_*K*_ = 30*n*^4^(*v* + 80), *I*_*l*_ = 0.1(*v* + 65), $I_{T}=0.5\,a_{\infty }^{3}\,r\,v$, $I_{\text{Ca}}=0.15\,s_{\infty }^{2}\,\left(v-120\right)$, *I*_AHP_ = 10×(*v* + 80)×*CA*/(*CA* + 10)
Gating kinetics	*w*_*m*_ = 37,σ_*m*_ = 10 λ_*h*_ = 0.05,*w*_*h*_ = 58,σ_*h*_ = −12,τ_*h*_ = 0.05 + 0.27/(1 + *exp*⁡((*v* + 40)/12)) λ_*n*_ = 0.1,*w*_*n*_ = 50,σ_*n*_ = 14,τ_*n*_ = 0.05 + 0.27/(1 + *exp*⁡((*v* + 40)/12)) λ_*r*_ = 1,*w*_*r*_ = 70,σ_*r*_ = −2,τ_*r*_ = 15 *w*_*a*_ = 57,σ_*a*_ = 2 *w*_*s*_ = 35,σ_*s*_ = 2 d*CA*/d*t* = 10^−4^×(−*I*_*Ca*_−*I*_*t*_−15×*CA*)

**TH**	

Ionic currents	*I*_Na_ = 3*m*^3^*h*(*v*−50), *I*_*K*_ = 5(0.75×(1−*h*))^4^(*v* + 75), *I*_*l*_ = 0.05(*v* + 70), $I_{T}=5\,p_{\infty }^{2}\,r\,v$
Gating kinetics	*w*_*m*_ = 37,σ_*m*_ = 7 λ_*h*_ = 1,*w*_*h*_ = 41,σ_*h*_ = −4,τ_*h*_ = 1/(0.128×*exp*⁡(−(*v* + 46)/18) + 4/(1 + *exp*⁡(−(*v* + 23)/5))) *w*_*p*_ = 60,σ_*p*_ = 6.2 λ_*r*_ = 1,*w*_*r*_ = 84,σ_*r*_ = −4,τ_*r*_ = 0.15×(28 + *exp*⁡(−(*v* + 25)/10.5))

**STR**	

Ionic currents	*I*_Na_ = 100*m*^3^*h*(*v*−50), *I*_*K*_ = 80*n*^4^(*v* + 100), *I*_*l*_ = 0.1(*v* + 67) *I*_*m*_ = *g*_*m*_*p*(*v* + 100)
Gating kinetics	α_*m*_ = 0.32×(54 + *v*)/(1−*exp*⁡(−(*v* + 54)/4)),β_*m*_ = 0.28×(27 + *v*)/(−1 + *exp*⁡((*v* + 27)/5)) α_*h*_ = 0.128×*exp*⁡(−(*v* + 50)/18),β_*h*_ = 4/(1 + *exp*⁡(−(*v* + 27)/5)) α_*n*_ = 0.032×(52 + *v*)/(1−*exp*⁡(−(*v* + 52)/5)),β_*n*_ = 0.5*exp*⁡(−(*v* + 57)/40) α_*p*_ = 3.209×10^−4^×(30 + *v*)/1−*exp*⁡(−(*v* + 30)/9) β_*p*_ = −3.209×10^−4^×(30 + *v*)/(1−*exp*⁡(−(*v* + 30)/9))

where *X* represented one of *a, b, c, d*1, *d*2, *h, m, n, p, q*, or *r*. Steady-state gating variables were calculated using


(3) $$X_{\infty }=\frac{1}{1+\exp\left(-\left(v+w_{X}\right)\,/\,\sigma_{X}\right)},$$

where *w*_*X*_ and σ_*X*_ were the half voltage and slope, respectively. Gating kinetics for STR took the form


(4) $$\frac{\text{d}\,X}{\text{d}\,t}=\alpha_{X}\,\left(1-X\right)-\beta_{X}\times X.$$

*I*_syn_ represented the sum of synaptic currents, with each projection from presynaptic neuron α to postsynaptic neuron β(α,β ∈ {*CTX*,*STR*,*STN*,*GP*,*TH*}) given by *I*_α→β_ = *g*_α,β_×(*v*_β_−*E*_syn_)×*S*, where *g*_α,β_ described the maximal synaptic conductance, and *E*_*syn*_ represented the reversal potential (uniformly set as −85 mV). An alpha synapse *S* was used to model the synaptic dynamics


(5) $$S=\frac{t-t_{d}}{\tau }\times {\text{e}}^{-\frac{t-t_{d}}{\tau }},$$

where *t*_*d*_ was the synaptic transmission delay, and τrepresented the time constant of 5 ms. Further, bias currents *I*_*app*_i_(*i* ∈ {*GPe*,*GPi*,*TH*}) represented other synaptic inputs that were not described explicitly in this model.

The dynamics of the CTX neurons were described based on the model developed by Izhikevich (2003)


(6) $$\begin{array}{c} \frac{\text{d}\,v_{\text{CTX}}}{\text{d}\,t}=0.04\times v_{\text{CTX}}^{2}+5\times v_{\text{CTX}}+140-u_{\text{CTX}}-I_{\text{interCTX}}-I_{\text{TH}\to \text{CTX}}\\ \frac{\text{d}\,u_{\text{CTX}}}{\text{d}\,t}=a\times \left(0.2\times v_{\text{CTX}}-u_{\text{CTX}}\right) \end{array}.$$

where *v*_CTX_ represented the transmembrane potential and *u*_CTX_ was the recovery variable. The time scale of the recovery variable *u*_CTX_ was chosen as *a* = 0.02 and *a* = 0.1, respectively, for excitatory-CTX (eCTX) and inhibitory-CTX (iCTX) neurons. If the transmembrane potential of CTX neuron exceeded 30 mV, then *v*_CTX_ was set to a resting potential equal to -65 mV and *u*_CTX_ was set to *u*_CTX_ + *d* (for eCTX, *d* = 8; for iCTX, *d* = 2). *I*_interCTX_ represented the reciprocal synaptic current from iCTX neurons to eCTX neurons or that from eCTX neurons to iCTX neurons, and *I*_TH→CTX_ was the synaptic input from TH.

The Parkinsonian state was simulated by the adjustment of model parameters implemented using a parkinsonism variable, *pd*, where *pd* = 0 and *pd* = 1 were defined as the healthy state and full Parkinsonian state, respectively. The M-type potassium current in striatal neurons was reduced*g*_m_ = 2.6−0.9×*pd*, cortico-striatal coupling strength was decreased *g*_*CTX*,*STR*_ = 0.07−0.044×*pd* and coupling strength between GPe neurons was increased*g*_*GPe*,*GPe*_ = 0.0125 + 0.0375×*pd*. To quantify the difference between the healthy and Parkinsonian states, changes of firing rates and firing patterns of model neurons were analyzed. It was considered that one neuron produced a spike or action potential once its transmembrane potential was greater than the threshold *V*_thre_ = −20mV, with the time of crossing the threshold defined as the firing time. The average firing rates were calculated based upon the firing time during the entire simulation period. In addition, spike synchrony that characterizes the dynamic patterns in each population of model neurons was measured. Defining *v*^*i*^(*t*) as the membrane potential time course of the *j*th neuron from a population of *n* neurons, then we could average over the population $V\,\left(t\right)=\frac{1}{n}\,\sum_{i=1}^{n}v^{i}\,\left(t\right)$. The variance of membrane potential *v*^*i*^(*t*) and the variance of the averaged membrane potential *V*(*t*) were expressed as $\sigma_{v_{i}}^{2}={\left\langle {\left[v_{i}\,\left(t\right)\right]}^{2}\right\rangle }_{t}-{\left[{\left\langle v_{i}\,\left(t\right)\right\rangle }_{t}\right]}^{2}$ and $\sigma_{V}^{2}={\left\langle {\left[V\,\left(t\right)\right]}^{2}\right\rangle }_{t}-{\left[{\left\langle V\,\left(t\right)\right\rangle }_{t}\right]}^{2}$, respectively $({\left\langle \cdots \right\rangle }_{t}=\frac{1}{T}\int_{0}^{T}\cdots \text{d}t$ referred to the average value of the variables within the time of T), and the level of synchrony was calculated according to the following equation,


(7) $$\chi =\sqrt{\frac{\sigma_{V}^{2}}{\frac{1}{n}\,\sum_{i=1}^{n}\sigma_{V_{i}}^{2}}},$$

where χ was normalized between 0 and 1, with χ = 0 indicating neurons within one population fire out of sync and χ = 1 indicating neurons within that population discharge synchronously.

### Control Problem Description

Parkinson’s disease is characterized by diverse changes in neuronal activity, and single neuron action potentials, electrocorticograms, LFPs, and electroencephalograms have been considered as feedback control signals for closed-loop DBS (Hoang et al., 2017). The LFP generated by model GPi neurons was adopted as the feedback signal for closed loop control. A simple average of transmembrane potentials was adopted to calculate the LFP of the modeled population due to its ability to capture subthreshold activity and thereby reflect oscillatory phenomena (Pettersen et al., 2012; Mazzoni et al., 2015). Expression of the GPi LFP was given as


(8) $$\text{LFP}\,\left(t\right)=\frac{1}{n}\,\sum_{i=1}^{n}v_{\mathrm{GPi}\,\_\,i}.$$

Here, *n* = 10 represented the total number of GPi neurons and *v*_*GPi*_i_ corresponded to the transmembrane potential of the *i*th GPi neuron. The power within particular frequency bands of the LFP signal was determined from power spectra using the Chronux neural signal analysis package [length of moving window 1 s, step size 0.1 s and tapers in the form of [3 5] (3 is the time-bandwidth product and 5 is the number of tapers to be used)], and the beta band power was defined as the total power over 13–30 Hz.

Our goal was to design an adaptive closed-loop controller to adjust automatically the stimulation signals delivered to the model neurons of the GPi based on the beta LFP activity calculated from the model GPi neurons as the feedback control signal. We defined the stimulation signal as *I*_sti_ and delivered it directly to each GPi neuron, and the resulting transmembrane potential was expressed as


(9) $$C\,\frac{\text{d}\,v_{\text{GPi}}}{\text{d}\,t}=-I_{\text{l}}-I_{\text{K}}-I_{\text{Na}}-I_{\text{T}}-I_{\text{Ca}}-I_{\text{ahp}}-I_{\text{STN}\to \text{GPi}}-I_{\text{GPe}\to \text{GPi}}-I_{\text{dSTR}\to \text{GPi}}+I_{\mathrm{app}\,\_\,\mathrm{GP}\,i}+I_{\text{sti}}.$$

*I*_sti_ was constructed by using the controller output *u*(*t*) to construct a variable frequency pulse train stimulation signal [amplitude of 300μ*A*/*cm*^2^, pulse duration of 0.3 ms and period of 1,000/*u*(*t*) ms (frequency of *u*(*t*))]. After the end of each stimulation period, we recalculated the beta power of the LFP signal and repeated the above steps to update continuously the optimal controller output. The transmembrane potentials of model neurons in that other nuclei were unaffected by direct electrical stimulation but their activity was influenced during stimulation via either excitatory or inhibitory synaptic connections.

The classical error-based PID control law has the form


(10) $$u_{\text{pid}}\,\left(t\right)=k_{\text{p}}\,e\,\left(t\right)+k_{\text{i}}\,\int e\,\left(t\right)\,\text{d}\,t+k_{\text{d}}\,\frac{\text{d}\,e\,\left(t\right)}{\text{d}\,t},$$

where *e*(*t*) = *y*_*d*_(*t*)−*y*(*t*)(*y*_*d*_(*t*) and *y*(*t*) represented the beta power in LFP signals of the GPi from healthy control and controlled Parkinsonian states, respectively). The performance of the PID controller depends greatly on selecting the appropriate gains, and this can be a time-consuming manual process. As the LFP was a highly dynamic variable subject to large changes, differential action might amplify noise interference. Therefore, the differential term was omitted, and both proportional (P) and proportional-integral (PI) controllers were designed to minimize the error between the desired and measured beta band power in the LFP.

**FIGURE 2:**
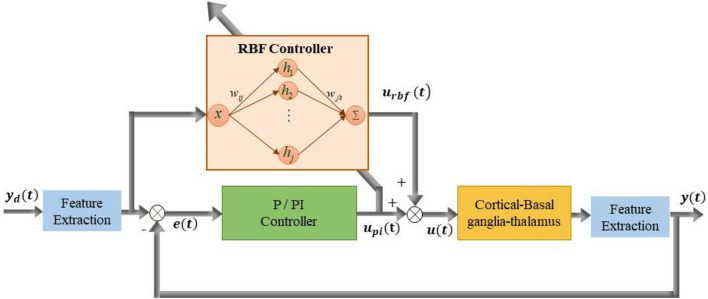
Block diagram of improved supervisory algorithm under the guidance of radial basis function (RBF) network. Stimulation signal *u*(*t*) is applied to GPi model neurons and the simulated beta power *y*(*t*) is obtained from LFP of the GPi. The stimulation signal *u*(*t*) is determined by the joint action of the P controller output *u*_p_(*t*) and RBF network controller output *u*_rbf_(*t*).

A stable self-tuning controller was designed using a dynamic RBF network. Figure 2 is a block diagram of the RBF supervisory control system and a schematic diagram of the RBF network. The neural network-based controller acted as a feedforward controller, by building an inverse model of the controlled plant. The input layer, hidden layer and output layer determined the structure of the RBF neural network. *x* = [*x*_1_,*x*_2_,…,*x*_*i*_,…,*x*_*n*_]^*T*^ (*n* was the number of input layer nodes) represented the network input vector and *h* = [*h*_1_,*h*_2_,…,*h*_*j*_,…,*h*_*m*_]^*T*^ (*m* was the number of hidden layer nodes) represented the hidden layer output. Each hidden layer node had a central value *c*_*j*_, the Euclidean distance of which to network input *x*_*i*_ was described as ||*x*_*i*_−*c*_*j*_||. As well, each hidden layer node was an arithmetic element with activation function given by


(11) $$h_{j}=\exp\left(-\frac{{||x-c_{j}||}^{2}}{2\,b_{j}^{2}}\right).$$

The center vector *c* = [*c*_1_,*c*_2_,…,*c*_*m*_] and the width vector *b* = [*b*_1_,*b*_2_,…,*b*_*m*_]^*T*^ determined the influence of the Gaussian function, where the width of the Gaussian basis function directly influenced the mapping capability to network input, while the center value correlated with its sensitivity to network input.

If we set the weight vector as *w* = [*w*_1_,*w*_2_,…,*w*_*m*_]^*T*^, then the network output can be obtained as


(12) $$u_{r\,b\,f}\,\left(t\right)=h_{1}\,w_{1}+\cdots +h_{j}\,w_{j}+\cdots +h_{m}\,w_{m}.$$

The structure of the RBF network was selected as 1−*n*_*m*_−1, that is, containing 1 input layer node, *n*_*m*_ hidden layer nodes and 1 output layer node. The number of hidden layer nodes was set to 11, network weights were initially set to random values between 0 and 1, and parameters of Gaussian function were set as *c* = [−2,−1,0,1,2]^*T*^, *b* = [5,5,5,5,5]^*T*^. The weights of the RBF controller were continuously adjusted on-line to make the feedback error *e*(*t*) approach zero, which equated to *u*_p_(*t*) approaching zero. Consequently, the RBF controller gradually occupied the leading position and even replaced the function of the P/PI controller. The RBF network error index was designed in the form of $E\,\left(t\right)=\frac{1}{2}\,{\left(u_{\text{p}}\,\left(t\right)\right)}^{2}$, to lead *u*_p_(*t*)(*e*(*t*)) to converge to 0. Considering that the total controller output was the sum of the traditional P/PI controller and the adaptive RBF controller *u*(*t*) = *u*_rbf_(*t*) + *u*_p_(*t*), the error index can be written as


(13) $$E\,\left(t\right)=\frac{1}{2}\,{\left(u_{\text{rbf}}\,\left(t\right)-u\,\left(t\right)\right)}^{2}$$

According to the gradient descent method, the network weights were adjusted as follows


(14) $$\Delta \,w_{j}\,\left(t\right)=-\eta \,\frac{\partial E\,\left(t\right)}{\partial w_{j}\,\left(t\right)}=-\eta \,\left(u_{\text{rbf}}\,\left(t\right)-u\,\left(t\right)\right)\,h_{j}\,\left(t\right),$$

(15) w⁢(t)=w⁢(t-1)+Δ⁢w⁢(t)+α⁢(w⁢(t-1)+w⁢(t-2)).

Further, applying the gradient descent method to the adjustment of *c* and *b* will optimize effective learning by the RBF network, thus we had


(16) $$\Delta \,b_{j}\,\left(t\right)=-\eta \,\frac{\partial E}{\partial b_{j}}=-\eta \,\left(u_{\text{rbf}}\,\left(t\right)-u\,\left(t\right)\right)\,w_{j}\,h_{j}\,\frac{||{\text{x-c}}_{j}^{2}||}{b_{j}^{3}},$$

(17) b⁢(t)=b⁢(t-1)+Δ⁢b⁢(t)+α⁢(b⁢(t-1)+b⁢(t-2)),

(18) $$\Delta \,c_{j}\,\left(t\right)=-\eta \,\frac{\partial E}{\partial c_{j}}=-\eta \,\left(u_{\text{rbf}}\,\left(t\right)-u\,\left(t\right)\right)\,w_{j}\,h_{j}\,\frac{x-c_{j}}{b_{j}^{2}},$$

(19) c⁢(t)=c⁢(t-1)+Δ⁢c+α⁢(c⁢(t-1)-c⁢(t-2)).

where η ∈ (0,1) represented the learning rate η = 0.30and α ∈ (0,1) represented the momentum factor α = 0.05.

A quantitative index of the control effect was defined as the root mean square error between the controlled output and the reference signal,


(20) $$\text{RMSE}=\sqrt{\frac{1}{N}\,\sum_{i=1}^{N}{\left(y_{i}-y_{d}\right)}^{2}}$$

where *N* represented the sampling point of the feedback signal.

## Results

The biophysically-based cortical-Basal-thalamus network model was used to test the effectiveness of closed-loop DBS. Performance of the RBF network-based supervisory algorithm was evaluated by considering changes in the state of the plant intended to represent dynamics including cycling of medication and progress of the disease, as well as dynamic changes in the reference (target) signal.

### Firing Rates and Firing Patterns of Model BG Neurons

**FIGURE 3:**
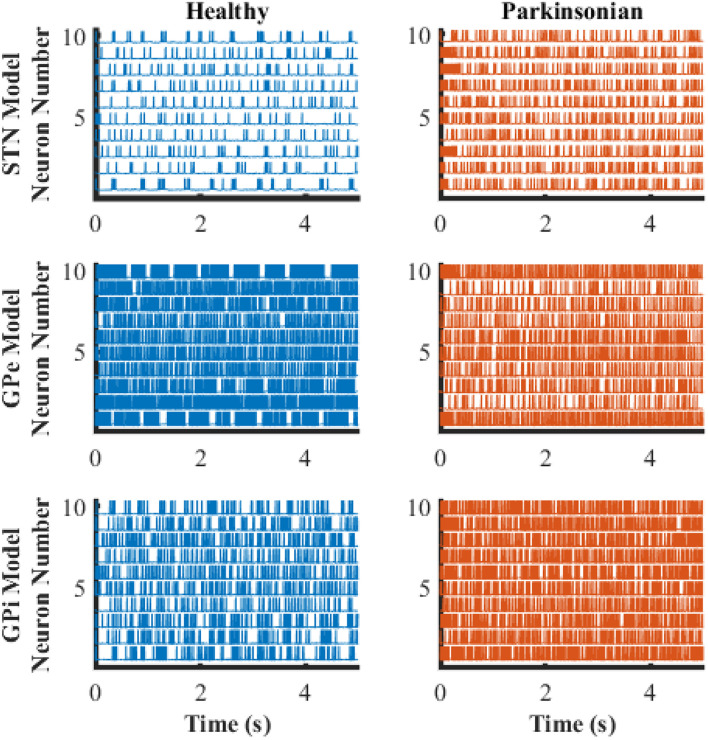
Transmembrane potential as a function of time of model neurons in STN, GPe, and GPi. The blue traces depict model neuron activity in the healthy condition while orange traces depict model neuron activity in the Parkinsonian condition.

**FIGURE 4:**
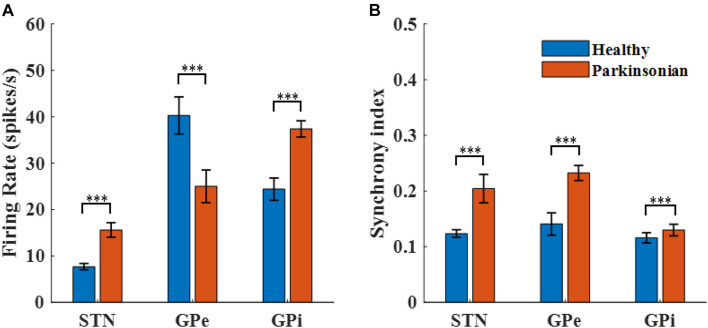
Characterization of model neuron activity in healthy and Parkinsonian conditions. **(A)** Firing rates (mean ± standard error) for model STN, GPe, and GPi neurons in healthy (blue) and Parkinsonian (orange) conditions. Values are averaged across three runs for each nucleus. **(B)** Spike synchrony (mean ± standard error) for model STN, GPe, and GPi neurons under healthy (blue) and Parkinsonian (orange) conditions. All model neurons exhibit increases in spike synchrony in the Parkinsonian state as compared to the healthy state. (*** represented a significant difference, *p* < 0.001).

The transmembrane potentials of model GPe, GPi, and STN neurons in the cortical-Basal-thalamus network model are displayed in Figure 3. The Parkinsonian condition resulted in changes in both the rate and pattern of model neuron activity. As a result of excitation via the indirect pathway and hyper-direct pathway together with inhibition via the direct pathway, firing rates in the Parkinsonian condition increased in STN and GPi model neurons and decreased in GPe model neurons (Figure 4A), consistent with previous experimental studies (Kita and Kita, 2011). Moreover, increased spike synchrony was observed across all nuclei in the Parkinsonian state compared to the healthy state (Figure 4B). These indexes were analyzed using one-way analysis of variance (ANOVA), which revealed a significant difference in firing rate (STN: *F* = 901 and *p* < 0.001, GPe: *F* = 48 and *p* < 0.001, and GPi: *F* = 184 and *p* < 0.001) and synchrony index (STN: *F* = 239 and *p* < 0.001, GPe: *F* = 1554 and *p* < 0.001, and GPi: *F* = 62 and *p* < 0.001) between healthy state and Parkinsonian state. The model thus exhibited features of the pathophysiological neural activity occurring in PD and was a suitable testbed to develop and analyze closed-loop control strategies.

**FIGURE 5:**
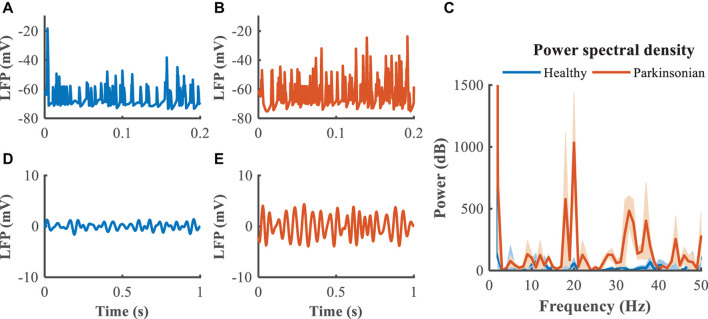
Local field potential (LFP) activity from model neurons in the GPi. In panels **(A,B)**, blue trace depicts the LFP in the healthy state while the orange trace depicts the LFP in the Parkinsonian state. Panel **(C)** illustrates the power spectral density of the GPi LFP across 10 trails to quantify the corresponding oscillatory activity, where shaded error region represents standard errors. Panels **(D,E)** depict the band-pass filtered (13–30 Hz) LFP activity in the GPi.

The GPi, which is clinically accessible for both recording of LFPs and delivery of DBS, was selected as the source of the feedback signal for closed loop DBS (Figures 5A,B). Compared to the healthy state, where the LFP exhibited little power in the beta band, the LFP in the Parkinsonian condition exhibited oscillatory activity, generating a significant peak in the power spectrum (Figure 5C). The LFP signals were filtered within the beta band to extract differences between healthy and Parkinsonian states, and the filtered GPi LFP activity in the healthy state (Figure 5D) served as a reference to guide the modulation of Parkinsonian neural activity by DBS, thus constituting a closed-loop system to suppress exaggerated beta oscillatory activity (Figure 5E).

### Limitation of Traditional P and PI Controllers

**FIGURE 6:**
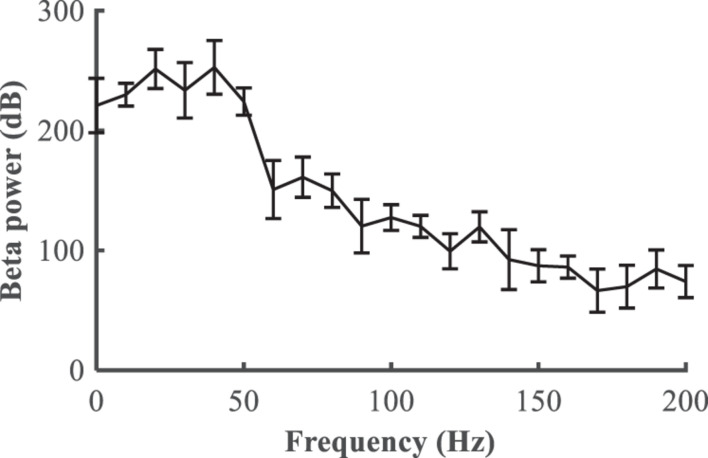
Relationship between the deep brain stimulation pulse repetition frequency and the beta power in the LFP from the GPi (mean ± standard error for 50 trials).

We first quantified the relationship between the stimulation frequency and the beta power (Figure 6), where *f* = 0 is equivalent to the Parkinsonian state without DBS. Low frequency stimulation actually increased beta power, and the beta power was progressively reduced as the stimulation frequency was increased higher than 50 Hz. A beta power of 120 dB was calculated from the healthy state and set as the desired state, and the range of stimulation frequencies was between 5 and 200 Hz. The modulation of DBS was reformulated as a train of monophasic pulses with an initial stimulation frequency of 5 Hz, and the updated stimulation frequency was generated based upon the error between the actual and target beta power in the LFP from the GPi model neurons.

**FIGURE 7:**
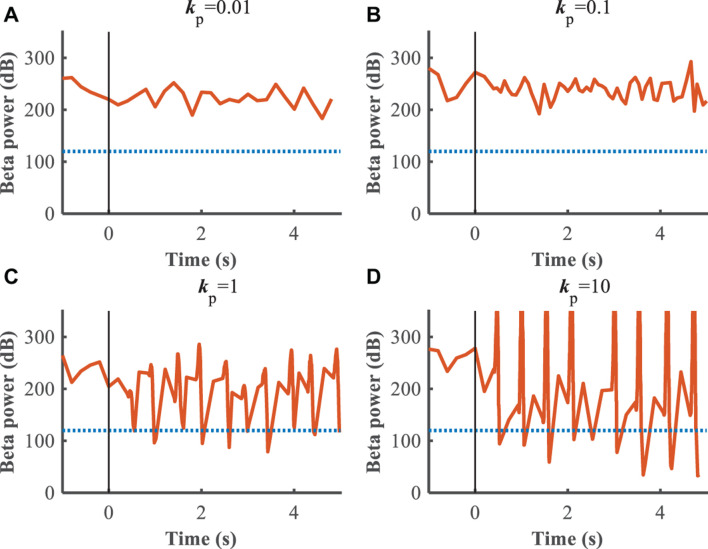
Control effect of P controller with beta power as the feedback signal. Blue dotted lines represent the desired GPi beta power that recorded from healthy state, and orange traces represent feedback GPi beta power from the controlled state. Black solid lines denote the beginning of stimulation. **(A)** *k*_p_ = 0.01, **(B)** *k*_p_ = 0.1, **(C)** *k*_p_ = 1, **(D)** *k*_p_ = 10.

**FIGURE 8:**
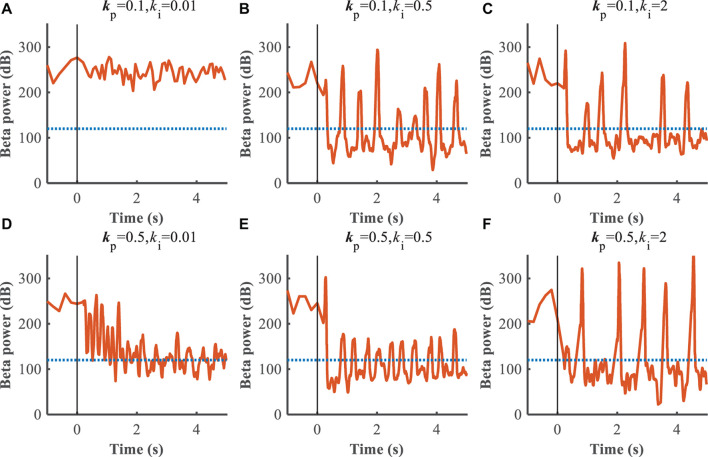
Control effect of PI controller with beta power as the feedback signal. Blue dotted lines represent the desired GPi beta power that recorded from healthy state, and orange traces represent feedback GPi beta power from the controlled state. Black solid lines denote the beginning of stimulation. **(A)** *k*_p_ = 0.1,*k*_i_ = 0.01, **(B)** *k*_p_ = 0.1,*k*_i_ = 0.5, **(C)** *k*_p_ = 0.1,*k*_i_ = 2, **(D)** *k*_p_ = 0.5,*k*_i_ = 0.01, **(E)** *k*_p_ = 0.5,*k*_i_ = 0.5, **(F)** *k*_p_ = 0.5,*k*_i_ = 2.

The performance of the P controller and PI controller in regulating the beta band LFP in the GPi with GPi DBS were compared (Figures 7, 8), where the blue dotted lines represented the healthy state and the orange traces represented the controlled PD state. Small *k*_p_ appeared to make no difference to the suppression of beta band activity, while large *k*_p_ caused strong oscillations between effective and ineffective suppression of beta band activity (Figure 7). The performance following addition of the integral controller with different combinations of *k*_p_ and *k*_i_ was assessed (Figure 8). The control of beta power in the GPi LFP was strongly dependent on the selection of *k*_p_ and *k*_*i*_, where effective values (*k*_p_ = 0.5,*k*_i_ = 0.5) promoted the suppression of high beta power while ineffective values (*k*_p_ = 0.1,*k*_i_ = 0.01) did not contribute to improvement of the PD state. The search of proportional and integral gains through trial-and-error produced fluctuations in performance and created uncertainty about the effectiveness of closed-loop DBS, especially in the face of changes in the properties of the plant.

**FIGURE 9:**
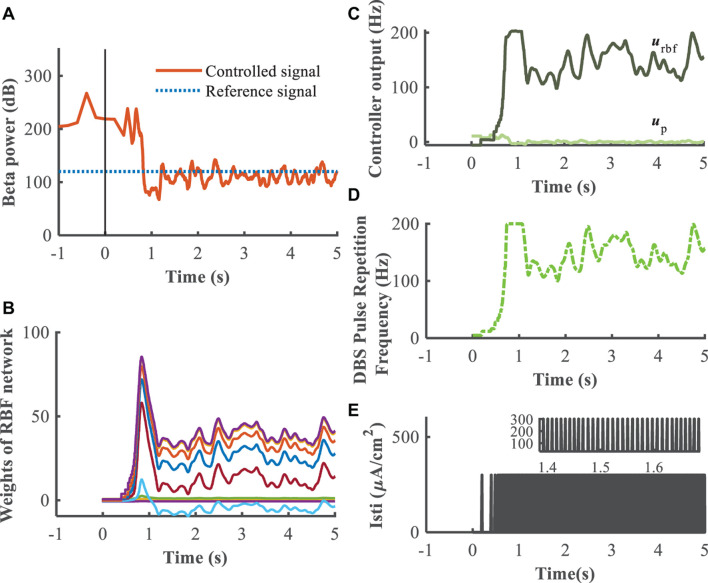
Feedback control using the RBF controller with beta power as the control signal (*k*_p_ = 0.1). Panel **(A)** depicts the dynamic process of the controller reducing beta power in the GPi, panel **(B)** shows the evolution of real-time updated weights of the RBF network, **(C)** plots the trend of P controller and RBF controller, respectively, **(D)** generates the DBS pulse repetition frequency. Panel **(E)** is the stimulation signal, a series of 0.3 ms duration 300μ*A*/*cm*^2^ amplitude pulses with the instantaneous frequency determined by the controller.

A substantial improvement in control performance was achieved by the supervisory algorithm, where suppression of the exaggerated beta power present in the Parkinsonian condition was achieved within 1 s (Figure 9A). The weights of the RBF network were adjusted in real-time, in response to the update of the beta power of the GPi (Figure 9B). As the beta power was gradually suppressed, the RBF network took over the leading role that the P/PI controller played in the initial control stage (Figure 9C). Ultimately, the DBS pulse repetition frequency was calculated as shown in Figure 9D, and the DBS signal is depicted in Figure 9E. Changes in beta power between the healthy state, open-loop 130 Hz DBS, P controller, PI controller and adaptive DBS were compared by calculating the root mean square error between the controlled output signal and the reference signal during the last 4 s of simulation. The averaged *RMSE* were 18.64, 112.41, 39.38, and 23.89, respectively, In cases of open-loop 130 Hz DBS, P control (*k*_p_ = 0.1), PI control (*k*_p_ = 0.5,*k*_i_ = 0.5) and combined P and RBF control, indicating that the improved supervisory algorithm drive the beta power to the target setting with a higher accuracy.

### Evaluation of Robustness of the Supervisory Algorithm

The closed-loop RBF network-based supervisory control algorithm achieved effective tracking of the target beta power, but several challenges required further consideration. First, Parkinson’s disease is a chronic and progressive disease in which the patient’s condition gradually worsens over time, and individual variation should be considered in model-based evaluations. Second, the reference waveform was the LFP signal from the healthy network model. Although this signal carries abundant information, it does not represent the variety of different disease states, for example during cycling of medication, and this potentially limits the generalizability of the control system.

**FIGURE 10:**
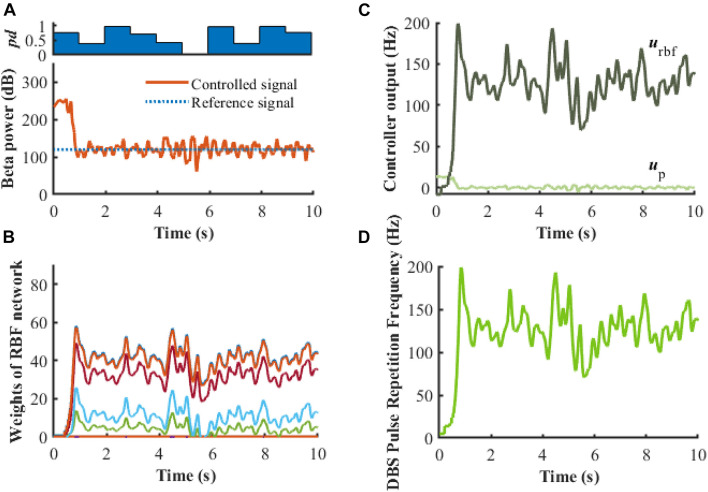
Robustness analysis of the RBF controller in the presence of dynamic changes in the Parkinsonian state (*k*_p_ = 0.1). **(A)** Dynamic change of Parkinsonian state is characterized by the parameter, *pd*. The RBF-controller modulated beta power during dynamic changes is depicted in the bottom panel. Panel **(B)** shows the evolution of real-time updated weights of the RBF network, **(C)** plots the trend of P controller and RBF controller, and **(D)** generates the DBS pulse repetition frequency. Here, the stimulation amplitude is set to 300μ*A*/*cm*^2^ and the pulse duration is set to 0.3 ms.

**FIGURE 11:**
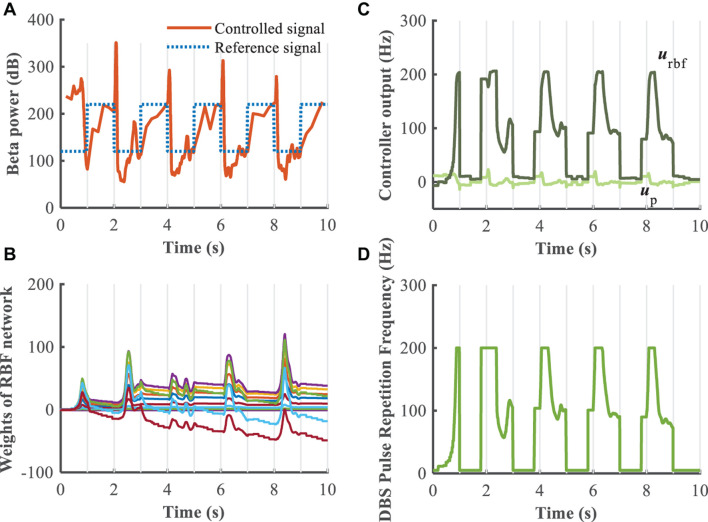
Robustness analysis of the RBF controller during dynamic changes in the reference beta power (*k*_p_ = 0.1). **(A)** Dynamic change of the reference beta power is characterized by the blue dotted line. The RBF-controller modulated beta power is depicted in the bottom panel. Panel **(B)** shows the evolution of real-time updated weights of the RBF network, **(C)** plots the trend of P controller and RBF controller, and **(D)** generates the DBS pulse repetition frequency. Here, the stimulation amplitude is set to 300μ*A*/*cm*^2^ and the pulse duration is set to 0.3 ms.

To evaluate further the performance of the proposed closed-loop algorithm in the face of changes in the Parkinsonian state (Figure 10), the parkinsonism variable *pd* was randomly generated from 0 to 1. Controlled beta power gradually converged to the desired healthy signal after 1 s, demonstrating the adaptive capability of the RBF network across disease states. In addition, beta power exhibits dynamic changes, especially prior to and during movement, and thus tracking of time-varying beta power may be required to promote desired movement behavior. In the face of a time varying reference beta power signal switching at 1 Hz (Figure 11), the controlled beta power still followed the dynamic reference signal, albeit with substantial overshoot.

## Discussion

This manuscript proposed an improved supervisory control algorithm for adaptively adjusting the stimulation signal to improve DBS control of the Parkinsonian state. Myriad control algorithms have been applied to the design of closed-loop DBS system, for example, on-off control, dual-threshold control, delayed feedback control, PID control, fuzzy control, and model predictive control (Little et al., 2013; Liu et al., 2015, 2016; Popovych et al., 2017; Su et al., 2019; Velisar et al., 2019). For on-off control and dual-threshold control, a stimulus was triggered by the control signal exceeding or falling below a threshold. Energy consumption was reduced as compared to traditional open-loop stimulation, but selection of optimal stimulation parameters during the DBS-on stage still needed to be addressed. The design of delayed feedback controller or PID controller depended strongly on the selection of controller gains and delay time constant, and the performance was dependent on the plant. Thus, such controllers may exhibit limited adaptability for individual variations due to, for example, changes in medication status or active versus inactive state. More advanced control algorithms, for example fuzzy control and model predictive control, have been developed for modulation of DBS parameters. However, the robustness of the control algorithm was improved at the cost of using a non-standard signal–the unmodulated controller output was applied directly to the stimulated targets, and this may be difficult to implement with an implanted pulse generator. In addition, Gao et al. (2020) proposed a deep reinforcement learning-based approach to construct an adaptive DBS framework. The reinforcement signal provided by the environment was an evaluation of the quality of the action that the agent produced. It should be noted that the external environment yielded evaluations (reward or punishment) rather than correct answers to the output of the learning system, and the performance of the learning system was improved by reinforcing the actions that were rewarded. Neural networks have the capacity to approximate arbitrary complex nonlinear systems, and an RBF network was adopted in this manuscript for selecting the appropriate pulse repetition frequency of DBS. The proposed RBF-based algorithm constituted supervised learning that provided a corresponding target output for each input. Through the feedback structure, the stability and robustness can be guaranteed, and the precision and adaptability were improved.

A biophysiologically feasible cortex-basal ganglia-thalamus computational network model that represents the Parkinsonian state in 6-OHDA lesioned rats was used as the plant and to calculate LFP signals and the effects of DBS. DBS of the STN or the GPi are currently the most common and effective surgical targets for the treatment of PD, but there does not appear to be one superior target. Several studies compared the efficacy of stimulating STN versus GPi and that both STN-DBS and GPi-DBS are equally effective in improving motor dysfunction (Honey et al., 2017; Zhang et al., 2021). STN-DBS contributes to more significant medication reduction and is favorable to decrease energy consumption due to the smaller stimulating region, but STN-DBS appears to increase the incidence of psychiatric complication. If medication reduction is not a major concern, GPi-DBS has the advantage of direct dyskinesia suppression. LFP signals from the GPi, which can be directly obtained through DBS leads in clinical application (Stanslaski et al., 2012; Arlotti et al., 2016b; Parastarfeizabadi and Kouzani, 2019) and carry abundant potential information from synchronous neural activity were extracted and processed as the feedback signal for closing the loop (Little and Brown, 2012; Priori et al., 2012; Hebb et al., 2014; Arlotti et al., 2016a; Sinclair et al., 2018). The design of the closed-loop control system followed a traditional strategy. For such a highly nonlinear and complex plant, the selection of optimal proportional gains was challenging, and simulation results illustrated that the P controller did not achieve effective tracking of the reference signal. The RBF neural network exhibits both self-learning and self-adaptation and was the foundation for constructing an intelligent control system. The improved supervisory control algorithm with the RBF network controller showed satisfactory tracking performance and was able to regulate the beta oscillatory power across dynamic changes in the plant and the reference signal.

The proposed algorithm has several potential advantages for clinical implementation. First, although the closed-loop control algorithm was designed based on a biophysical model of the cortex-basal ganglia-thalamus network, precise parameters (e.g., synaptic conductance, reversal potentials) and network structure (e.g., synaptic connectivity) were not necessary since the RBF network builds an inverse representation based on input output information. Second, DBS stimulation signals were delivered through and LFP recordings were obtained from same implanted electrode, thereby avoiding the requirement of additional external sensors. A limitation of this simulation study is the setting of the desired tracking signal, and the variable dynamics of the cortical-Basal-thalamus network were not fully considered. Further exploration combined with the selection of biological markers that relate to specific symptoms and states remains an important challenge. Further, understanding the relationship between stimulation parameter changes and changes in specific patient symptoms, including the time course of such changes, is crucial to improving clinical treatment. For example, data-driven input-output model identification might be a promising solution for quantifying responsiveness to specific stimulation signals.

## Data Availability Statement

The original contributions presented in the study are included in the article/supplementary material, further inquiries can be directed to the corresponding author.

## Author Contributions

YZ was responsible for manuscript development, concept, and study design, and wrote the manuscript. WG conceived the project, and supervised the study design and manuscript development. JW collaborated in manuscript development and concept. HL and CL collaborated in methodology design and implementation. All authors read and approved the final manuscript.

## Conflict of Interest

The authors declare that the research was conducted in the absence of any commercial or financial relationships that could be construed as a potential conflict of interest.

## Publisher’s Note

All claims expressed in this article are solely those of the authors and do not necessarily represent those of their affiliated organizations, or those of the publisher, the editors and the reviewers. Any product that may be evaluated in this article, or claim that may be made by its manufacturer, is not guaranteed or endorsed by the publisher.
